
JOURNAL INFORMATION
==============================
NLM Title Abbreviation: Am J Trop Med Hyg
Journal ID: Am J Trop Med Hyg
NLM Journal ID: 0370507
Journal ID: tpmd

The American Journal of Tropical Medicine and Hygiene

ISSN: 0002-9637
EISSN: 1476-1645
Publisher: The American Society of Tropical Medicine and Hygiene

ARTICLE INFORMATION
==============================
PMCID: PMC11376120
DOI: 10.4269/ajtmh.1113stoc
Article ID: tpmd1113stoc
Article version: 1
Subjects: Other

Contents

Electronic publication date: 2024 Sep 3
Print publication date: 2024 Sep
Volume: 111
Issue: 3 Suppl
Issue title: A Legacy of Impact in Global Health: Tribute to President Jimmy Carter and Mrs. Rosalynn Carter
First page: ii
Last page: ii
Copyright: © 2024, The American Society of Tropical Medicine and Hygiene
Copyright year: 2024
License: This is an open-access article distributed under the terms of the Creative Commons Attribution (CC-BY) License, which permits unrestricted use, distribution, and reproduction in any medium, provided the original author and source are credited.
License URL: https://creativecommons.org/licenses/by/4.0/

Page count: 1

==============================

### A Legacy of Impact in Global Health: Tribute to President Jimmy Carter and Mrs. Rosalynn Carter

**Guest Editor: Mark Eberhard**

### CONTENTS

**A Legacy of Impact in Global Health**
Mark L. Eberhard 1

**Evaluation of the International Task Force for Disease Eradication: A Review of Past Deliberations**
Shanze Sadiq, Ursula A. Kajani, Anyess R. Travers, Donald R. Hopkins, Frank Richards, and Kashef Ijaz 5

**Slaying the Serpent: A Research Agenda to Expand Intervention Development and Accelerate Guinea Worm Eradication Efforts**
Maryann G. Delea, Alexandra Sack, Obiora A. Eneanya, Elizabeth Thiele, Sharon L. Roy, Dieudonne Sankara, Kashef Ijaz, Donald R. Hopkins, and Adam J. Weiss 12

**Predicting the Environmental Suitability and Identifying Climate and Sociodemographic Correlates of Guinea Worm (*Dracunculus medinensis*) in Chad**
Obiora A. Eneanya, Maryann G. Delea, Jorge Cano, Philip Ouakou Tchindebet, Robert L. Richards, Yujing Zhao, Abdalla Meftuh, Karmen Unterwegner, Sarah Anne J. Guagliardo, Donald R. Hopkins, and Adam Weiss 26

**Factors Influencing Community Engagement during Guinea Worm and Polio Eradication Endgames in Chad: Recommendations for "Last Mile" Programming**
Maryann G. Delea, Lalique Browne, Severin Kaji, Adam J. Weiss, and Ouakou Tchindebet 36

**Serological Responses to Trachoma Antigens prior to the Start of Mass Drug Administration: Results from Population-Based Baseline Surveys, North Darfur, Sudan**
Angelia M. Sanders, Balgesa E. Elshafie, Zeinab Abdalla, Courtney Simmons, Erica Brook Goodhew, Tania A. Gonzalez, Andrew W. Nute, Atif Mohammed, Elizabeth Kelly Callahan, Diana L. Martin, and Scott D. Nash 49

**Integrated Serosurveillance for Onchocerciasis, Lymphatic Filariasis, and Schistosomiasis in North Darfur, Sudan**
Jenna E. Coalson, Gregory S. Noland, Andrew W. Nute, Erica Brook Goodhew, Diana L. Martin, Zeinab Abdalla, Isam Zarroug, Soheir Gabralla, Hassan Ahmed Hassan Ahmed Ismail, William Evan Secor, Elizabeth Kelly Callahan, Angelia M. Sanders, Balgesa Elshafie, and Scott D. Nash 58

**A Mixed-Methods Evaluation of Mainstreaming Mass Drug Administration for Schistosomiasis and Soil-Transmitted Helminthiasis in Four Districts of Nigeria**
Emily Griswold, Abel Eigege, Emmanuel C. Emukah, Jayden Pace Gallagher, Jenna Coalson, Lindsay Rakers, Bulus Mancha, Okocha Ndudi, Paul Ugbadamu, Philomena Dikedi, Happiness Poko, Jacob Danboyi, Philemon Dagwa, Vincent Anighoro, Christiana Davou Gwong, Esther Otabor, Goodluck James Amayat, Regina Ese Unukopia, Emmanuel S. Miri, and Gregory S. Noland 69

**Results of Integrated Transmission Assessment Surveys for Lymphatic Filariasis and Malaria in Haiti, 2017–2022**
Luccène Désir, Karen E. S. Hamre, Valéry Madsen Beau De Rochars, Jean F. Lemoine, Marc-Aurèle Telfort, and Gregory S. Noland 81

**Over 25 Years of Hope: Development of Lymphatic Filariasis Patient Support Groups in Haiti**
Valery Madsen Beau De Rochars, Jeannine Coreil, Martha Désir, Gladys Mayard, Marie Carmel Michel, Marie Denise Milord, Rand Carpenter, Thomas G. Streit, Luccène Désir, Gregory S. Noland, and David G. Addiss 89

**Assessing Stigma Toward Mental, Neurological, and Substance Use Disorders in Liberia: A Population Representative Study**
S. Benedict Dossen, J. Mike Mulbah, April Hargreaves, Samhita Kumar, David Mothersill, Gerard Loughnane, Eve Byrd, Angie Tarr Nyakoon, Joseph S. Quoi, and Ikenna D. Ebuenyi 93

**The Epidemiology of Ocular *Chlamydia trachomatis* Infection within Districts Persistently Endemic for Trachoma in Amhara, Ethiopia**
Scott D. Nash, Eshetu Sata, Ambahun Chernet, Tania A. Gonzalez, Andrew W. Nute, Victoria C. Ontiveros, Demelash Gessese, Mulat Zerihun, Kimberly A. Jensen, Gizachew Yismaw, Taye Zeru, Berhanu Melak, Zebene Ayele, Fetene Mihretu, Fikre Seife, Zerihun Tadesse, and E. Kelly Callahan 105

**Impact of a School Trachoma Program Emphasizing Facial Cleanliness and Environmental Improvement in Amhara, Ethiopia**
Caleb D. Ebert, Ayenachew Kerie, Melke Kifle, Scott D. Nash, Zerihun Tadesse, Abebe Fissha, Berhanu Melak, Kassa Bulcha, Melak Haileleule, Awoke Dagnew, Ewnetu Bazie, Mitiku Adugna, Elizabeth Kelly Callahan, Mulaw Abebe, Kimberly A. Jensen, and Eshetu Sata 114

**Severity of Trachomatous Scarring among Adults in Trachoma-Endemic Amhara Region of Ethiopia**
Jaymie A. Bromfield, Ugochi T. Aguwa, Eshetu Sata, Kimberly A. Jensen, Fetene Mihretu, E. Kelly Callahan, Sheila K. West, Meraf A. Wolle, and Scott D. Nash 121

**Scorecard Approach to Eliminate Onchocerciasis in Venezuela**
Oneida Camacho, Carlos Botto, Dalila Ríos, Benny Barrios, Sharly Ceballos, Oscar Noya-Alarcón, Miguel Fernández, Yseliam Tovar, Nestor J. Villamizar, Lindsay Rakers, Frank Richards, and María Eugenia Grillet 127

**Progress Toward Onchocerciasis Elimination in Brazil**
João Luiz Pereira de Araujo, Dalila Ríos, Maria Eugenia Grillet, Alda Maria da Cruz, Lindsay Rakers, Frank Richards, Heriberto Francis Schuertz, and Sandra Maria Barbosa Durães 137

**Do Residents Believe that Onchocerciasis Transmission Was Eliminated? Results of A Post-Treatment Surveillance Knowledge, Attitude, and Practices Survey in Three Foci of Uganda**
Lauri Bernard, Annet T. Khainza, Edson Byamukama, Moses Katabarwa, Jenna Coalson, Gregory S. Noland, and Frank O. Richards Jr. 141

For more information about
the American Society of Tropical Medicine and Hygiene
please visit us at www.astmh.org

The American Journal of Tropical Medicine and Hygiene is
online at www.ajtmh.org
